# Neuroprotection for Age-Related Macular Degeneration

**DOI:** 10.1016/j.xops.2022.100192

**Published:** 2022-07-05

**Authors:** Jonathan B. Lin, Yusuke Murakami, Joan W. Miller, Demetrios G. Vavvas

**Affiliations:** 1 Ines and Frederick Yeatts Retina Research Laboratory, Retina Service, Department of Ophthalmology, Massachusetts Eye and Ear, Harvard Medical School, Boston, Massachusetts; 2 Department of Ophthalmology, Graduate School of Medical Sciences, Kyushu University, Fukuoka, Japan

**Keywords:** Age-related macular degeneration, Neuroprotection, Retinal degeneration, AD, Alzheimer disease, ALA, alpha lipoic acid, AMD, age-related macular degeneration, AREDS, Age-Related Eye Disease Study, AREDS2, Age-Related Eye Disease Study 2, *CFH*, complement factor H, CNTF, ciliary neurotrophic factor, GA, geographic atrophy, *HTRA1*, high-temperature requirement A1, IOP, intraocular pressure, iPSC, induced pluripotent stem cell, RBP, retinol-binding protein, RGC, retinal ganglion cell, RIPK3, receptor-interacting serine/threonine-protein kinase 3, ROS, reactive oxygen species, RPE, retinal pigment epithelium, VA, visual acuity

## Abstract

Age-related macular degeneration (AMD) is a leading cause of blindness worldwide. Early to intermediate AMD is characterized by the accumulation of lipid- and protein-rich drusen. Late stages of the disease are characterized by the development of choroidal neovascularization, termed “exudative” or “neovascular AMD,” or retinal pigment epithelium (RPE) cell and photoreceptor death, termed “geographic atrophy” (GA) in advanced nonexudative AMD. Although we have effective treatments for exudative AMD in the form of anti-VEGF agents, they have no role for patients with GA. Neuroprotection strategies have emerged as a possible way to slow photoreceptor degeneration and vision loss in patients with GA. These approaches include reduction of oxidative stress, modulation of the visual cycle, reduction of toxic molecules, inhibition of pathologic protein activity, prevention of cellular apoptosis or programmed necrosis (necroptosis), inhibition of inflammation, direct activation of neurotrophic factors, delivery of umbilical tissue–derived cells, and RPE replacement. Despite active investigation in this area and significant promise based on preclinical studies, many clinical studies have not yielded successful results. We discuss selected past and current neuroprotection trials for AMD, highlight the lessons learned from these past studies, and discuss our perspective regarding remaining questions that must be answered before neuroprotection can be successfully applied in the field of AMD research.

Age-related macular degeneration (AMD) is a leading cause of blindness worldwide. Therapies that inhibit VEGF have been revolutionary for patients with the exudative or wet form of advanced AMD. Nonetheless, anti-VEGF therapies have no role in treating advanced nonexudative or dry advanced AMD or patients with exudative AMD who respond to anti-VEGF treatments but ultimately develop macular atrophy. Thus, there is a significant clinical need to develop novel treatments to slow or prevent photoreceptor death.

The pathogenesis of advanced AMD is complex. Past studies relying on cellular and animal models with confirmatory studies in humans have suggested that there are numerous pathways involved in AMD pathogenesis, including but not limited to altered lipid homeostasis, dysregulated immune activation, increased oxidative stress, and impaired metabolism.^1^ Despite significant efforts, there is no clear consensus regarding the predominant causative pathologic process or processes that ultimately lead to photoreceptor death. It is becoming increasingly apparent that AMD is a multifactorial disease whose pathogenesis likely involves perturbations in numerous molecular and cellular pathways that ultimately lead to photoreceptor degeneration. Because photoreceptors are the light-sensitive cells of the retina that are responsible for phototransduction, photoreceptor death inevitably causes blindness.

Neuroprotection has emerged as a strategy for delaying photoreceptor death and preserving vision. One advantage of the broad strategy of neuroprotection is that it may have the ability to slow photoreceptor degeneration regardless of the underlying causative pathway and may even be generalizable to other retinal neurodegenerative diseases. For the purposes of this review, we define “neuroprotection” to be any therapeutic strategy intended to enhance photoreceptor survival, regardless of whether they target a primary causative or secondary/contributory pathologic process. Moreover, we focus on pharmacologic strategies rather than nonpharmacologic interventions, such as laser. Other therapeutic strategies, such as those intended to inhibit pathologic complement activation, which likely involve other retinal cells, have been reviewed.^2^ In this review, we highlight selected past and ongoing clinical studies investigating neuroprotection as a strategy for treating AMD, provide our perspective regarding the lessons learned from these studies, and discuss remaining areas of important research that must be explored before neuroprotection can become a reality for AMD therapy.

## Past and Current Neuroprotection Trials

**Figure 1:**
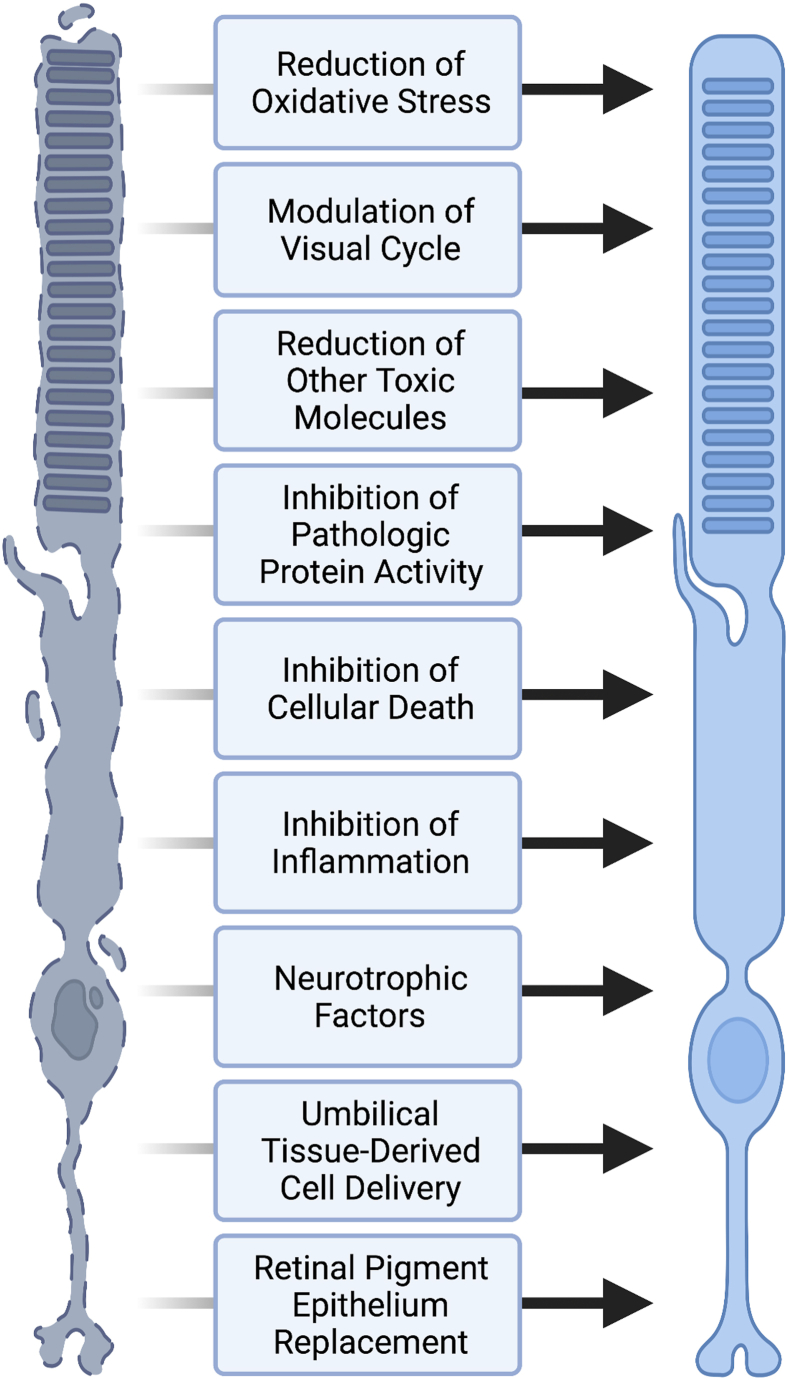
Strategies for neuroprotection in age-related macular degeneration (created with BioRender.com).

There are numerous past and ongoing neuroprotection trials in AMD. Broadly, these approaches can be divided into their overall strategy: reduction of oxidative stress, modulation of the visual cycle, reduction of toxic molecules, inhibition of pathologic protein activity, inhibition of cellular death pathways such as apoptosis or programed necrosis (necroptosis), inhibition of inflammation, direct activation of neurotrophic factors, delivery of umbilical tissue–derived cells, and retinal pigment epithelium (RPE) replacement (Fig 1). Selected trials are highlighted in Table 1.

**Table 1:** Selected Ongoing and Completed Neuroprotection Trials for Age-Related Macular Degeneration

Strategy	Drug Name (Company)	Target	Delivery Route	Current Status
Reduction of Oxidative Stress	AREDS2 supplements (vitamin D, vitamin E, lutein, zeaxanthin, and zinc)	Antioxidant supplements	Oral	Widely in use
	Alpha lipoic acid	Antioxidant supplement	Oral	Intolerable, no effect in RCT
	Vitamin D	Antioxidant (?)	Oral	No effect in RCT
	Omega-3 fatty acids	Antioxidant (?)	Oral	No effect in RCT
	OT-551 (Othera Pharmaceuticals)	Terminate free radical reactions	Eye drops	Failed in Phase II
	Elamipretide (Stealth BioTherapeutics)	Enhance mitochondrial function and reduce ROS	Subcutaneous	Failed in Phase II
	Risuteganib (Allegro Ophthalmics)	Regulate integrins	Intravitreal	Completed Phase IIa
Modulation of Visual Cycle	Fenretinide (ReVision Therapeutics)	RBP antagonist	Oral	Failed in Phase II
	LBS-008 or tinlarebent (Belite Bio)	RBP4 antagonist	Oral	Completed Phase I
	Emixustat (Kubota Pharmaceutical Holdings)	Inhibit RPE65	Oral	Failed in Phase IIb/III
	ALK-001 (Alkeus Pharmaceuticals)	C20 deuterated vitamin A	Oral	Now in Phase III
Reduction of Toxic Molecules	GSK933776 (GlaxoSmithKline)	Anti-beta-amyloid antibody	Intravenous	Failed in Phase II
	RN6G (Pfizer)	Anti-beta-amyloid antibody	Intravenous	Terminated in Phase II
	GAL-101 (Galimedix Therapeutics)	Prevent formation of toxic beta-amyloid oligomers	Eye drops	Passed Phase I
Inhibition of Pathologic Protein Activity	FHTR2163 (Genentech)	Anti-*HTRA1* antibody	Intravitreal	Now in Phase II
Prevention of Apoptosis	ONL1204 (ONL Therapeutics)	Inhibit Fas	Intravitreal	Now in Phase I
Inhibition of Inflammation	Doxycycline	Tetracycline antibiotic with anti-inflammatory effects	Oral	Completed Phase II/III without results
	Sirolimus	Inhibit mTOR	Intravitreal and subconjunctival	Failed in Phase I/II
Neurotrophic Factors	NT-501 (Neurotech Pharmaceuticals)	Deliver CNTF	Intravitreal implant	Completed Phase II
	Brimonidine Drug Delivery System (Brimo DDS; Allergan, an AbbVie company)	Alpha-2-adrenergic agonist	Intravitreal depot	Now in Phase III
	AL-8309B (Alcon)	Serotonin A1 receptor agonist	Eye drop	Failed in Phase III
	Iluvien (Alimera Sciences)	Deliver fluocinolone	Intravitreal depot	Terminated in Phase II
Human Umbilical Tissue–Derived Cell Delivery	Palucorcel or CNTO-2467 (Janssen Pharmaceuticals)	Deliver human umbilical tissue–derived cells	Subretinal	Failed in Phase II
RPE Replacement	OpRegen (Lineage Cell Therapeutics)	Replace RPE	Cell transplantation	Now in Phase I/IIa
	ASP7317 (Astellas)	Replace RPE	Cell transplantation	Now in Phase I
	CPCB-RPE1 (Regenerative Patch Technologies)	Replace RPE	Monolayer transplantation	Now in Phase I/II
	PF-05206388 (Moorfields Eye Hospital)	Replace RPE	Monolayer transplantation	Now in Phase I

AREDS2 = Age Related Eye Disease Study 2; CNTF = ciliary neurotrophic factor; *HTRA1* = high-temperature requirement A1; mTOR = mammalian target of rapamycin; RBP = retinol binding protein; RCT = randomized controlled trial; ROS = reactive oxygen species; RPE = retinal pigment epithelium.

## Reduction of Oxidative Stress

### Systemic Natural Antioxidants

One neuroprotective approach is to reduce exposure of photoreceptors to oxidative stress. The initial evidence that targeting oxidative stress may protect against advanced AMD was from the Age-Related Eye Disease Study (AREDS). In AREDS, post-hoc analysis of a specific cohort of patients with intermediate AMD was found to have reduced odds of progression to advanced neovascular AMD if they were randomized to the combination of numerous antioxidants (vitamin C, vitamin E, beta-carotene) plus zinc compared with those randomized to placebo.^3^, ^4^, ^5^, ^6^ The subsequent Age-Related Eye Disease Study 2 (AREDS2) replaced beta-carotene with other antioxidants, lutein and zeaxanthin, because beta-carotene was found to be associated with increased rate of lung cancer among smokers in large population studies. There was no difference in the rate of progression to advanced AMD in those with the new or old formulation.^7^ The AREDS2 supplements are widely used in patients with intermediate nonexudative AMD, although the durability and magnitude of effect may be small, and this initial finding has never been replicated in a subsequent randomized placebo-controlled phase III trial.

Other systemic antioxidants, such as alpha lipoic acid (ALA), have been tested as well. However, high-dose ALA was found to be intolerable in older subjects because of flushing sensation and gastrointestinal side effects.^8^ Moreover, a randomized clinical trial showed that ALA had no beneficial effect on GA or visual acuity (VA) in patients with advanced nonexudative AMD.^9^ Forms of lipoylcarnitine esters may be more bioavailable and more suitable for testing in AMD.

A prespecified ancillary study of the Vitamin D and Omega-3 Trial showed that randomization to vitamin D and omega-3 fatty acids, which are thought to have possible antioxidative properties, did not lead to any significant reduction in either AMD incidence or AMD progression.^10^

### Local Antioxidants

Local delivery of antioxidants to the retina has also been tested. This strategy has the theoretical benefit of increased bioavailability to the eye with fewer systemic adverse effects. OT-551 (Othera Pharmaceuticals) is a lipophilic, disubstituted hydroxylamine that is administrated as an eye drop. Both OT-551 and its active metabolite, TEMPOL-H, can terminate free radical reactions in vitro and thereby exert an antioxidant effect with the potential result of protecting photoreceptors from oxidative damage. Although OT-551 was safe and tolerable when dosed 4 times daily for up to 2 years, it did not reduce GA growth or reduce the extent of VA loss in patients with AMD.^11^^,^^12^

Another agent that has shown promise is elamipretide (Stealth BioTherapeutics), a peptide that enhances mitochondrial function and reduces formation of reactive oxygen species (ROS) in vitro. Elamipretide is being investigated for numerous diseases, especially those in which mitochondrial dysfunction is suspected to play a pathogenic role. The Phase 1 ReCLAIM study suggested that subcutaneous elamipretide is tolerable and may improve visual function in patients with noncentral GA or those with high-risk drusen.^13^^,^^14^ These initial results have led to the Phase II ReCLAIM-2 trial (NCT03891875). Unfortunately, the drug failed on the primary endpoints of low-luminance VA and GA progression, though the secondary endpoints of potentially less ellipsoid zone loss was encouraging to the study investigators.

The small peptide integrin regulator risuteganib (Allegro Ophthalmics), which reduces ROS and enhances mitochondrial function in preclinical studies, has also shown promise. In a recent Phase IIa study, patients randomized to risuteganib had significant improvement in VA compared with control patients.^15^ We should be cautious in interpreting these successes given the inherent limitations of early-phase studies.

## Modulation of the Visual Cycle

The visual cycle is necessary for regeneration of functional visual pigment for phototransduction. Over a lifetime, continuous activity of the visual cycle can lead to accumulation of by-products such as N-retinylidene-N-retinylethanolamine, a bisretinoid that is suspected to have a cytotoxic effect and contribute to photoreceptor degeneration in diseases such as AMD. Accordingly, another neuroprotective strategy for AMD is modulation of the visual cycle, thereby reducing formation of toxic by-products.

Fenretinide (ReVision Therapeutics) is a retinol-binding protein (RBP) antagonist. Retinol-binding proteins are essential for transporting vitamin A in the systemic circulation. Retinol-binding protein antagonism can reduce systemic vitamin A availability and, thus, reduces activity of the visual cycle by limiting substrate availability. Despite this theoretical effect, oral fenretinide was not found to have any significant effect on GA lesion growth in a Phase II clinical trial.^16^ Use of fenretinide has also been associated with impaired dark adaptation and dyschromatopsia, both in this study in AMD patients and in the oncology literature.^16^^,^^17^ Other inhibitors of specific RBPs, such as LBS-008 (also known as “tinlarebant”; Belite Bio), an antagonist of RBP4, are still under investigation. In 2020, Belite Bio announced positive safety results from their Phase I clinical trial of LBS-008 and are planning to initiate studies investigating the potential role of LBS-008 for treating dry AMD and Stargardt disease.

Another strategy for modulating the visual cycle has been attempted with emixustat (Kubota Pharmaceutical Holdings), a small-molecule inhibitor of retinoid isomerohydrolase (RPE65), an enzyme of the visual cycle. Although initial studies were promising, the subsequent Phase IIb/III SEATTLE study did not show any significant effect of emixustat on GA growth rate.^18^^,^^19^ Similar to patients taking fenretinide, a large proportion of patients on emixustat have delayed dark adaption and dyschromatopsia.^19^^,^^20^

Finally, the approach of disrupting the visual cycle via administration of a chemically modified form of vitamin A, ALK-001 (Alkeus Pharmaceuticals), has been attempted. This C20 deuterated vitamin A forms toxic by-products more slowly than normal vitamin A and thereby leads to fewer cytotoxic effects. ALK-001 has shown promising results for treating the inherited retinal degenerative disease, Stargardt disease, and is currently being tested for patients with AMD in the Phase III SAGA study (NCT03845582).

## Reduction of Other Toxic Molecules

Visual cycle by-products are not the only molecules that may cause cellular toxicity. The amyloid pathway involves cleavage of amyloid precursor protein into smaller fragments, including beta-amyloid. Accumulation of beta-amyloid in the brain has been shown to be neurotoxic in neurodegenerative diseases such as Alzheimer disease (AD), although the exact pathophysiological mechanism by which this occurs remains unclear. Beta-amyloid is also a component of drusen found in AMD patients and is known to activate the complement cascade.

In a Phase II study, GSK933776 (GlaxoSmithKline), an anti-beta-amyloid monoclonal antibody, was found to have no clinically meaningful effect on slowing VA loss or reducing GA growth.^21^ Phase II evaluation of another anti-beta-amyloid antibody, RN6G (Pfizer), was registered but terminated early because of an “organizational decision” (NCT01577381). The dipeptide GAL-101 (formerly known as MRZ-99030/EG30; Galimedix Therapeutics), which prevents formation of toxic beta-amyloid oligomers, has also been hypothesized to have a possible neuroprotective role for AMD. GAL-101 eye drops were shown to be safe and tolerable in patients with glaucoma in a Phase I study (NCT01714960). Although not yet registered, Galimedix Therapeutics reported plans to pursue a Phase II study of GAL-101 in patients with nonexudative AMD.

## Inhibition of Pathologic Protein Activity

High-temperature requirement A1 (*HTRA1*) is a trimeric serine protease that is widely expressed in the retina and known to cleave multiple substrates including extracellular matrix proteins. Its association with AMD was established on the basis of genetic studies showing an association between AMD and a locus on chromosome 10q26, which contains the *HTRA1* gene.^22^ Although it has not been definitively proven that the *HTRA1* gene is the gene within this locus that promotes AMD, *HTRA1* activity has been suggested to be pathologic and contribute to AMD pathogenesis based on preclinical studies. In line with this, intravitreal delivery of FHTR2163/RO7171009/RG6147, an anti-*HTRA1* antibody fragment (Genentech), was shown to be safe and well tolerated in Phase I^23^ and is now being evaluated as a therapy for GA in the Phase II GALLEGO study (NCT03972709).

## Inhibition of Cellular Death

**Figure 2:**
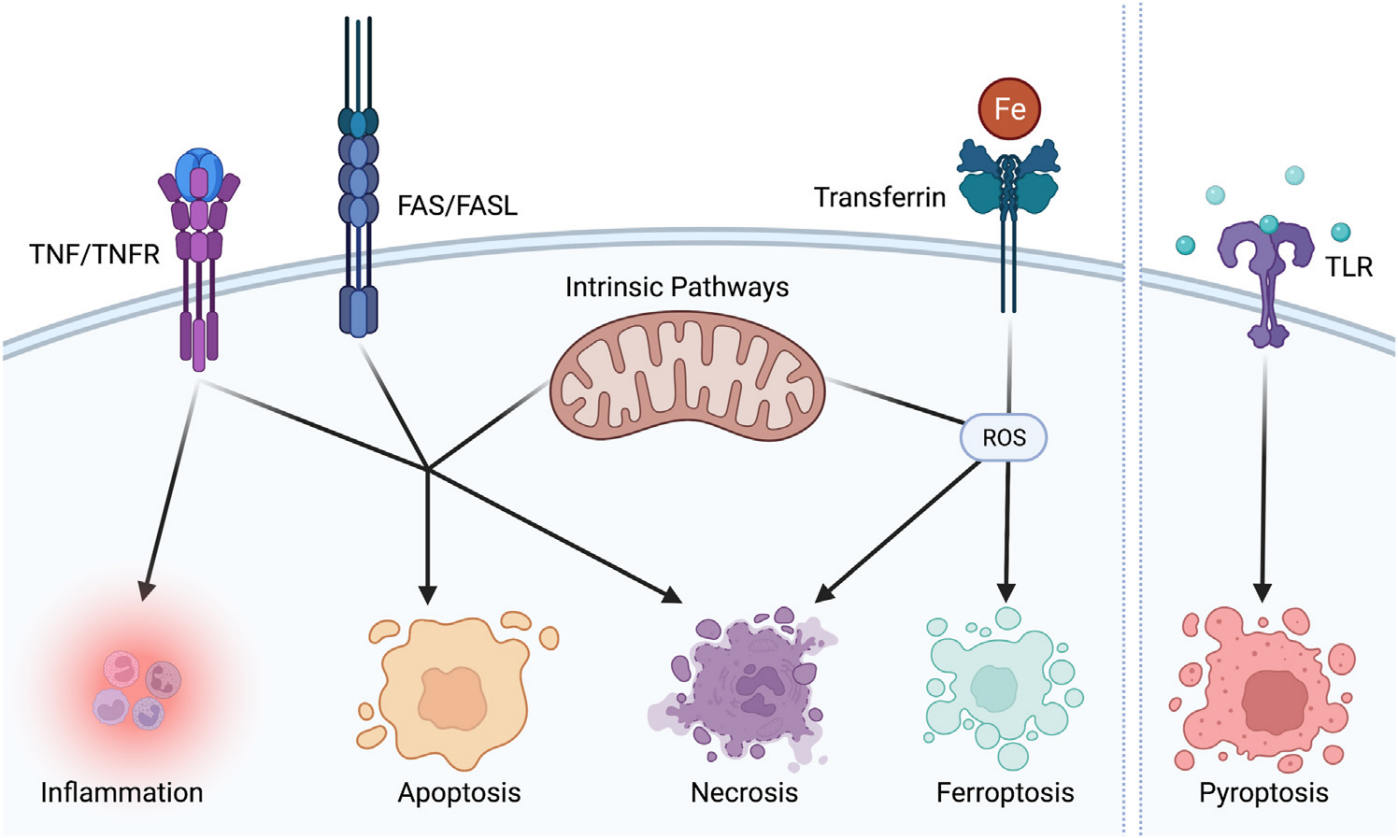
Numerous cellular death pathways can be triggered by diverse inciting stimuli, including activation of the tumor necrosis factor (TNF)/TNF receptor (TNFR) pathway, the FAS/FAS ligand (FASL) pathway, the iron (Fe)/transferrin pathway, the toll-like receptor (TLR), or intrinsic pathways. Activation of pyroptosis via the TLR pathway is best described in inflammatory cells, such as macrophages. ROS = reactive oxygen species (created with BioRender.com).

Another approach for preventing photoreceptor death is to inhibit activation of cellular death pathways, such as the Fas/Fas ligand interaction, which initiates a downstream cascade that ultimately triggers apoptotic cellular death or necroptotic cell death depending on the balance of complex intracellular signaling pathways.^24^, ^25^, ^26^, ^27^, ^28^, ^29^, ^30^, ^31^, ^32^ Intrinsic intracellular pathways can also trigger apoptosis or necrosis. Other cellular death pathways, such as ferroptosis and pyroptosis, have been speculated to play roles in photoreceptor degeneration. A schematic of cell death pathways is depicted in Figure 2. ONL1204 (ONL Therapeutics) is a small-molecule Fas inhibitor intended to protect photoreceptors from apoptosis triggered by pathologic pathways. Based on promising preclinical studies, a Phase I study testing its safety and tolerability in patients with GA is under way (NCT04744662).

Although further exploration in human studies is necessary, preclinical studies suggest that photoreceptors are affected by all these cell death pathways in a redundant and complementary fashion. There is complexity not only in that cone and rod photoreceptors may be affected by different predominant cell death pathways but also in that different cell death pathways may be triggered by different inciting stimuli. Thus, it may be necessary to target multiple death pathways simultaneously to achieve the desired clinical effect. Agents that target upstream initiators of cellular death pathways versus those that target end effectors have different advantages and disadvantages and need to be further explored.

## Anti-Inflammatory Agents

Preclinical studies suggest that inflammation may contribute to photoreceptor degeneration. Thus, some efforts have been directed toward evaluating possible anti-inflammatory agents for the purpose of neuroprotection. Although better known for its role as a tetracycline antibiotic, doxycycline (Oracea) is also used to treat inflammatory disorders such as rosacea. The Phase II/III TOGA study was intended to evaluate the possible role for doxycycline in slowing enlargement of GA area (NCT01782989); although this study was initiated in 2013 and was listed as completed as of November 2020, no results have yet been published. There have also been small Phase I/II studies investigating the possible role of intravitreal or subconjunctival delivery of sirolimus, an inhibitor of mammalian target of rapamycin, to slow GA. Intravitreal sirolimus was found to have possible adverse effects,^33^ whereas subconjunctival sirolimus was found to be well tolerated but without significant benefit.^34^

## Neurotrophic Factors

There are many endogenous molecules that are known to support the growth, survival, and differentiation of neurons, such as ciliary neurotrophic factor (CNTF), bone-derived neurotrophic factor, and glial cell line-derived neurotrophic factor, to name a few. These factors have been shown to be neuroprotective in numerous animal models of disease. As such, delivery of these factors may enhance photoreceptor survival in AMD.

NT-501/Renexus (Neurotech Pharmaceuticals) is an intraocular implant that delivers CNTF to the vitreous space via encapsulated cell technology. Subgroup analysis of an early study provided evidence that NT-501 may have a small benefit for some patients with GA.^35^ Notably, this beneficial effect on VA was seen only in patients with VA 20/63 or better at baseline. Because CNTF failed in neuroprotection trials for patients with retinitis pigmentosa,^36^ more recent studies are now focused on evaluating NT-501 for treating macular telangiectasia (NCT03316300, NCT03071965). It is also important to note that CNTF supplementation may have side effects in patients, such as miosis and suppressed electroretinogram responses, which may interfere with other aspects of visual function despite possibly promoting photoreceptor survival.

Other molecules have been found to have possible neuroprotective effect despite having a different primary effect. For example, brimonidine is an alpha-2-adrenergic agonist that is commonly prescribed for lowering intraocular pressure (IOP) in patients with glaucoma. Beyond its effect of lowering IOP, brimonidine is thought to be neuroprotective for retinal ganglion cells (RGCs) based on studies in animal models.^37^^,^^38^ A possible neuroprotective effect for brimonidine is also supported by findings of the Low-Pressure Glaucoma Treatment Study, in which the investigators found that despite achieving similar control of IOP, glaucoma patients randomized to brimonidine had less disease progression compared with patients randomized to a different IOP-lowering drop (timolol).^39^ One interpretation of this difference in effect, although not conclusive, is that brimonidine may also have a neuroprotective effect beyond its effect on IOP.

In view of this possibility, some investigators have tested whether brimonidine may also be neuroprotective for photoreceptors. The Brimonidine Drug Delivery System (Brimo DDS, Allergan, an AbbVie company) is an intravitreal implant designed as a depot formulation of brimonidine that can be administered via intravitreal injection and deliver brimonidine into the vitreous humor over a period of several months. A Phase II study in patients with GA found that there may be less progression in GA lesion area in patients randomized to Brimo DDS, although this difference was statistically significant only at month 3 and not at later time points.^40^ Despite limitations of this early study, such as insufficient statistical power, use of the last-observation-carried-forward method for missing data, and lower completion rate in the implant group (76%) versus the sham group (91%), further studies are under way.

In addition to their role in treating psychiatric illness, serotonin A1 receptor (5-HT1A) agonists are thought to have a potential neuroprotective role in the eye. Despite promising preclinical studies in animal models, the Phase III GATE study showed that AL-8309B/tandospirone (Alcon) eye drops had no beneficial effect on GA growth in patients with AMD.^41^

Another therapy that is well known for its other effects but may also have neuroprotective properties are corticosteroids. The fluocinolone acetonide intravitreal implant (Iluvien, Alimera Sciences) releases fluocinolone at a continuous low dose to the retina. Iluvien is approved for treatment of diabetic macular edema.^42^ A clinical trial examining the possibility that Iluvien may treat GA was registered but later terminated before completing enrollment (NCT00695318).

## Human Umbilical Tissue–Derived Cell Delivery

Another approach for neuroprotection is subretinal delivery of human umbilical tissue-delivered cells (palucorcel or CNTO-2467, Janssen Pharmaceuticals). Although the mechanism by which these cells protect photoreceptors remains under active investigation, preclinical models show promising results. Initial human studies showed an unacceptably high rate of retinal perforations and retinal detachments when delivering these cells to the subretinal space.^43^ The surgical technique has since been altered to make it safer, although the Phase II study still showed no significant decrease in GA area, retardation of GA growth rate, or improvement in VA in patients treated with palucorcel.^44^

## Retinal Pigment Epithelium Replacement

A different neuroprotective approach is based on the observation that RPE degeneration is observed in association with photoreceptor death in AMD. As such, some have hypothesized that partial restoration of the RPE may enhance photoreceptor resilience. OpRegen (Lineage Cell Therapeutics) is stem cell–based therapy that involves allogeneic RPE transplantation into the retina. It is currently being evaluated in a Phase I/IIa study (NCT02286089). Another therapy involving allogeneic RPE transplantation is ASP7317 (Astellas), which is also still in Phase I (NCT03178149). Other devices, including the CPCB-RPE1 (Regenerative Patch Technologies) and PF-05206388 (Moorfields Eye Hospital) implants, attempt to maintain the polarized orientation of RPE cells by immobilizing the RPE cells to a scaffold/membrane. These implants are in the early safety/tolerability phase of study (NCT02590692 and NCT01691261, respectively). Although still in their infancy, these strategies of RPE transplantation would be seminal if they are successful, because they would support the idea that RPE degeneration causes photoreceptor degeneration.

## Lessons Learned

Despite numerous attempts to achieve neuroprotection for AMD, many clinical studies have not been successful. Despite these disappointments, there are many lessons that can be gleaned from these past studies with numerous remaining questions to be addressed by further research. Key points that we have learned from past research are summarized in Table 2.

**Table 2:** Key Points Identified by Past Research on AMD

1. AMD is a complex, multifactorial disease that likely involves contributions from multiple pathologic processes that lead to photoreceptor death and blindness.
2. Cell-based models have their value but have inherent limitations because of their inability to capture the complexity of the human retina.
3. Rodent animal models, although helpful, have major limitations in their ability to model human disease given their lack of macula in addition to being rod photoreceptor-dominant, nocturnal animals.
4. Anti-VEGF agents have taught us that multiple diseases with heterogeneous underlying etiologies can be managed successfully with a common approach if they share a critical, common process.
5. Lessons from diverse neurodegenerative diseases, including beyond the eye, may be applicable to AMD and may lead unifying therapies that have widespread effects.

AMD = age-related macular degeneration.

## AMD as a Complex, Multifactorial Disease

There have been extensive investigations intended to better understand the pathologic mechanisms involved in AMD. Cumulatively, these studies have revealed numerous complex cellular and molecular processes that contribute to RPE and photoreceptor dysfunction, ultimately culminating in irreversible cell death and blindness. Despite numerous attempts to achieve neuroprotection with various strategies, many of these trials have not been fruitful. These failures support the notion that AMD is a complex, multifactorial disease, whose progression likely involves multiple pathophysiological processes that, in synergy, lead to observed cellular and tissue dysfunction.

One remaining question is the temporal relationship between RPE dysfunction and photoreceptor degeneration. This understanding would better guide treatments toward efforts to maintain RPE function or to preserve photoreceptor survival. Histopathological studies examining postmortem eyes from patients with GA have suggested that photoreceptor loss, especially rod photoreceptors, is observed early in absence of obvious morphological changes in Bruch’s membrane or RPE.^45^ In contrast, others speculate that accumulation of lipids and injury to RPE may precede photoreceptor degeneration.^46^^,^^47^ Further studies are necessary to clarify this relationship.

## The Value of Cellular and Animal Models

Past and current clinical trials are based on preclinical studies relying on cellular and animal models. Although in vitro and in vivo systems have many advantages, these systems are models of human disease and rarely recapitulate all features of a disease’s complex pathogenesis. Models of AMD are no exception. The failures to translate many preclinical studies of AMD into novel therapeutics may reflect the imperfections of our present models.

One facile approach for studying AMD pathogenesis is using in vitro models. Many studies have evaluated factors involved in photoreceptor and RPE survival by using immortalized cell lines, such as 661W cells^48^ or ARPE-19 cells.^49^ These cell lines proliferate rapidly, allowing for ease of experimentation. However, these immortalized cell lines recapitulate only some features of their corresponding epithelial cell type. As such, findings at the in vitro level do not always translate to the tissue or organismal level. Another approach has been to study RPE cells derived from induced pluripotent stem cells (iPSCs) or iPSC-RPE cells. Because iPSC-RPE cells can be generated from adult somatic cells of patients with ocular diseases, they allow for analysis at the patient-level to elucidate how gene mutations contribute to disease pathophysiology, a valuable tool, especially for inherited retinal degenerative diseases.^50^ One major limitation of all in vitro models is that they fail to account for the retina’s highly specialized structure. The complex interactions between photoreceptors and other retinal neurons with supporting cells, including RPE and choroid, are impossible to recapitulate in vitro.

Animal models, especially mouse models, are relied on extensively in biomedical science, and research in AMD is no exception. In contrast with in vitro models, these tools have the advantage of being able to model tissue- and organismal-level pathology. However, to date, there is no mouse model that develops all features of AMD in a progressive, stepwise fashion, resembling the disease process in human patients. Instead, there have been multiple mouse models identified that recapitulate certain features of AMD, such as development of (1) drusen or drusen-like deposits, (2) changes in Bruch’s membrane, (3) immune dysregulation in the retina, (4) oxidative damage in the retina, (5) photoreceptor and RPE cell death resembling GA, and (6) choroidal neovascularization. The details of these murine models, many of which involve genetic mutations and environmental factors, are the subject of other excellent reviews.^51^^,^^52^

**Figure 3:**
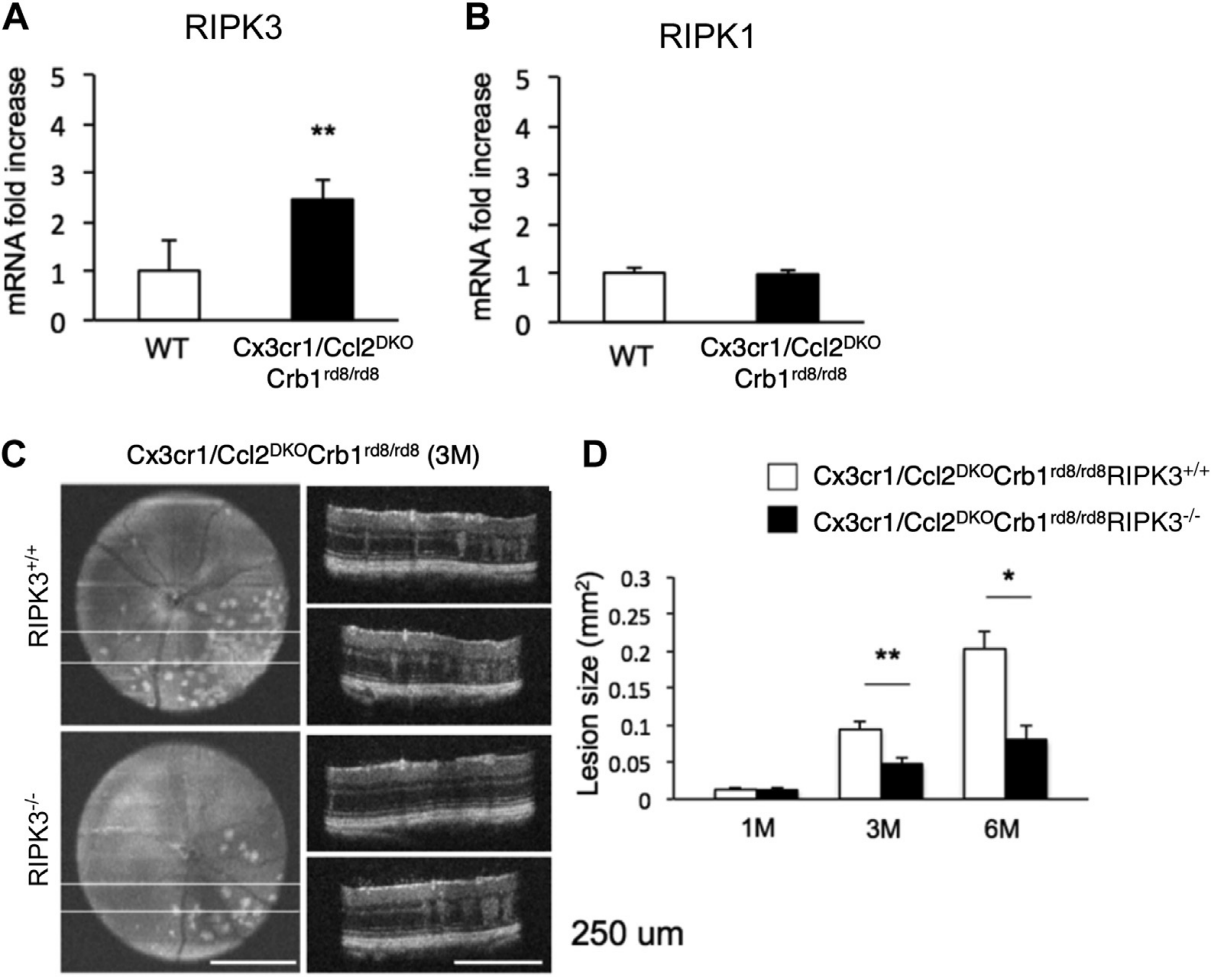
Necroptosis may drive retinal pathology in a mouse model of AMD-like pathology. **A,** Mice lacking the genes for the chemokine receptor Cx3cr1 and the chemokine Ccl2 on a background strain with the rd8 mutation of the *Crb1* gene (*Cx3cr1/Ccl2*^*DKO*^*Crb1*^*rd8/rd8*^; DKO = double knockout) have higher retinal messenger RNA (mRNA) expression of receptor-interacting serine/threonine-protein kinase 3 (RIPK3) compared with wild-type (WT) mice, **(B)** whereas there was no difference in expression of RIPK1. **C, D,***Cx3cr1/Ccl2*^*DKO*^*Crb1*^*rd8/rd8*^ mice that also have the gene for RIPK3 deleted have fewer focal lesions in the retina compared with *Cx3cr1/Ccl2*^*DKO*^*Crb1*^*rd8/rd8*^ mice that have intact RIPK3 at 3 months (3M) and 6 months (6M) of age (**C,** representative images with quantification of independent images in D). Graphs show mean + standard error of the mean **(A, B, D)**. ∗*P* < 0.05, ∗∗*P* < 0.01.

As an example of how animal models can still be used to help our understanding of human disease despite their limitations, we previously studied mutant mice lacking the genes for the chemokine CCL2 and the chemokine receptor CX3CR1 on a genetic background with the rd8 mutation of the *Crb1* gene (*Cx3cr1*^*-/-*^*Ccl2*^*-/-*^*Crb1*^*rd8/rd8*^). With age, these mice develop focal lesions and photoreceptor degeneration, which has now been shown to be due primarily to the rd8 mutation of the *Crb1* gene rather than *Cx3cr3/Ccl2* deletion.^53^^,^^54^ We found that these mutant mice had higher gene expression of receptor-interacting serine/threonine-protein kinase 3 (RIPK3) in the retina compared with mice with the normal/wild-type *Crb1* gene (Fig 3A), whereas there was no difference in gene expression of RIPK1 (Fig 3B). Receptor-interacting serine/threonine-protein kinase 1 and RIPK3 are known to be important for initiating the cell death pathway known as necroptosis. Moreover, when we also delete the gene coding for RIPK3 from these mice, there is significantly less accumulation of retinal pathology at 3 and 6 months of life (Fig 3C, D). Taken together, within the limitations of this model, our findings suggest that necroptosis may be involved in driving retinal pathology in mice and, thus, may have a role in photoreceptor death in AMD. These findings provide support for further investigation of necroptosis as a possible contributor to AMD pathology.

Other groups have also identified mouse models that recapitulate aspects of AMD pathobiology. Work by Toomey et al^55^ and Landowski et al^56^ highlighted the multifactorial contributions of immune regulation, diet, and age on retinal pathology. Prior genetic-wide association studies revealed a strong association between the *Y402H* variant of the gene for complement factor H (*CFH*), a regulator of the alternative complement pathway, and AMD.^57^ Bowes Rickman's group identified that mice with monoallelic *CFH* deletion or with expression of the H402 human pathologic variant of *CFH* develop drusen-like, retinal deposits at advanced age when challenged with a high-fat diet. The contribution of impaired cholesterol homeostasis to AMD pathology was further supported by seminal work by the Apte and Grimm labs: mutant mice lacking the cholesterol efflux transporters ATP-binding cassette transporter A1 and G1 specifically from macrophages, photoreceptors, or RPE all develop lipid-rich retinal deposits and associated retinal/RPE dysfunction mimicking AMD.^58^, ^59^, ^60^ These mouse models highlight a possible role for immune dysregulation and lipid dyshomeotasis in AMD pathogenesis and may have utility in future studies to test novel therapeutic strategies.

Although in vivo models have their value, the fact that they exhibit single features of disease in isolation challenges how relevant they may be for understanding the disease process in humans. Moreover, differences in retinal anatomy and structure when comparing humans with model organisms such as mice, including differences in types of photoreceptors (i.e., rod vs. cone photoreceptors) present in the retina, the underlying spatial distribution of the photoreceptors (i.e., presence or absence of fovea), and their nocturnal versus diurnal lifestyles, further complicate matters. Identification of new models or improvements of current models may be necessary to improve our understanding of complex AMD pathogenesis.

## The Right Drug for the Right Patient

Clinical classification systems for AMD have been developed for large clinical studies, such as AREDS.^61^ These classification systems imply that AMD progresses stepwise, although not necessarily linearly. However, it remains possible that AMD is not a monolithic disease. In fact, given the highly heterogeneous clinical presentation and clinical features of AMD, there may be multiple subtypes of AMD with different prognoses and underlying pathologic processes. This complexity has been highlighted by large population studies showing that patients with AMD typically have a combination of multiple genetic risk factors along with lifestyle factors influencing propensity for disease.^62^ Advanced imaging techniques such as swept-source OCT and OCT angiography may be able to provide further insight regarding inter-individual differences by identifying subclinical features of disease and may inform identification of AMD subclassifications.^63^

Another avenue for distinguishing subpopulations of patients with AMD who may respond differently to different therapies center around identifying biomarkers. As mentioned earlier, significant efforts have been undertaken to identify genetic mutations associated with AMD.^22^^,^^64^^,^^65^ This research has identified multiple loci within genes coding for proteins with diverse functions. Because certain genetic mutations may be associated with a less robust response to anti-VEGF treatments or to antioxidant/vitamin supplementation,^5^^,^^66^ it follows that patients with different underlying genetic mutations may have different underlying pathophysiology and thus might require different treatments for optimal management. Moreover, given a possible contribution of inflammation and immune dysregulation in AMD pathogenesis, others have focused on identifying inflammatory biomarkers, including both general serum markers of inflammation and markers of complement activation.^57^^,^^67^ Finally, because dysregulation of lipid metabolism is also thought to contribute to AMD pathogenesis, others have investigated whether patients with AMD also have systemic perturbations in lipid homeostasis.^68^^,^^69^ Given significant between-study heterogeneity related to both inflammatory and lipid biomarkers, more research is necessary to elucidate the clinical implications of the published findings. Nonetheless, we speculate that using biomarkers will likely be necessary for guiding precision-medicine efforts to tailor AMD treatments to a patient’s underlying pathophysiology. The myriad biomarkers of AMD that have been investigated are reviewed extensively by Lambert et al.^70^

## The Right Drug at the Right Time

Despite promising results from preclinical studies for numerous drugs that target various pathways thought to be involved in AMD pathogenesis, many of these drugs have failed to show efficacy in slowing GA progression in human clinical trials. One explanation for these failures is a true lack of clinical efficacy of the drugs. However, it remains possible that they are being tested in patients at too late a stage of AMD. In other words, it is possible that these drugs may have improved efficacy if they were used in early to intermediate AMD before the development of GA, although this poses a challenge because not all patients with early or intermediate AMD progress to advanced disease. Further studies are necessary not only to test this hypothesis but also to identify the characteristics of patients who may be at higher risk of progression to advanced disease and thus may benefit from early therapies.

## Lessons Learned from Other Cells

The use of neuroprotective strategies has been explored extensively in other fields, including in neurodegenerative diseases within the central nervous system, such as for AD, Parkinson’s disease, and neurodegeneration of other retinal cells, such as in glaucoma. We are not aware of any blockbuster successes in neuroprotection to date, but research in these other areas may have parallels to efforts in AMD that warrant consideration.

Work in the field of AD research may provide some lessons regarding properties of an ideal neuroprotective therapy for AMD. The hallmark pathological feature of AD is accumulation of extracellular plaques composed of beta-amyloid and intracellular neurofibrillary tangles containing tau. Although there are multiple pathologic pathways involved in AD pathogenesis, 2 key contributors are amyloid precursor protein mis-metabolism leading to amyloid deposition and tau hyperphosphorylation leading to tau misfolding and propagation.^71^ As a result, anti-amyloid and anti-tau strategies have been explored to reduce the extent of plaque and neurofibrillary tangle accumulation and possibly to slow onset of or to delay dementia. Nonetheless, despite significant efforts including numerous randomized clinical trials, none of these strategies have been successful to date. Of note, many of these therapies have succeeded in achieving their intended effect of reducing plaque or tau accumulation but without the patients having any clinical benefit. The anti-amyloid beta antibody aducanumab (Aduhelm; Biogen) was recently approved by the Food and Drug Administration under an accelerated pathway, but it was approved amid significant controversy and has not been widely implemented, with some even calling for the Food and Drug Administration to withdraw its approval.^72^, ^73^, ^74^ Because many insurance companies have refused to cover its cost, and the Centers for Medicare and Medicaid Services announced that they would cover its cost only in qualifying clinical trials, Biogen recently withdrew its marketing application for aducanumab in Europe.

These failures bring to the forefront the idea that solely treating one pathological feature of disease may not be sufficient to achieve a desired clinical effect. Although they have different underlying compositions, drusen, the sine qua non of AMD, are like beta-amyloid plaques in that they accumulate with age but are found at much higher concentrations in patients with disease. The failures of prior trials in AD suggest that therapies that solely target drusen biogenesis may not be sufficient to prevent vision loss in patients with AMD. This phenomenon has been observed in the past with prior trials demonstrating that laser photocoagulation of drusen leads to their regression but without significant reduction in risk of developing vision loss.^75^ Moreover, it is important to use patient-centered end points in future clinical trials in addition to molecular/imaging end points to ensure that novel therapies achieve not only a statistically significant but also a clinically significant benefit.

Work in the field of Parkinson’s disease may be informative in guiding how to approach neuroprotection in AMD. Parkinson’s disease is a disease of progressive loss of dopamine-producing neurons in the substantia nigra, leading to cardinal signs of resting tremor, bradykinesia, rigidity, and postural instability. The exact cellular mechanisms leading to neuronal death remain elusive, with some proposing that alpha-synuclein misfolding, mitochondrial dysfunction, impaired protein clearance, and neuroinflammation may contribute.^76^^,^^77^ Because the exact cause of neuronal death has not been established, current therapies center around enhancing dopaminergic signaling by administering dopamine precursors, dopamine agonists, or agents that slow dopamine metabolism. These therapies control motor symptoms but are not disease modifying insofar as they do not address the pathologic process causing dopaminergic neuron death and have limited durability.^78^ Applying these lessons to AMD, we speculate that a successful treatment for AMD will likely have to address the underlying pathologic process or even multiple pathologic processes to achieve the desired effect. Moreover, given growing lifespans as a result of innovations in health care, it will be increasingly important to consider the long-term durability of treatments as well.

Basic research in the field of glaucoma also offers some insight. By using single-cell RNA sequencing, some investigators have identified that there are subpopulations of RGCs that are more vulnerable to degeneration in mouse models of axonal degeneration.^79^ These vulnerable RGC subpopulations have unique genetic profiles. Beyond identifying novel molecular targets for neuroprotection, these findings may be relevant in that there may be photoreceptor subpopulations that are uniquely vulnerable. Finally, if there are common pathways involved in neurodegeneration of different tissues, it may be possible to develop unifying strategies for neuroprotection that can be used for multiple neurodegenerative diseases.

## Our Perspective

One major limitation of the neuroprotection clinical trials to date is that they provide limited insight into the primary or causative cellular death pathways in AMD. Although there is not yet clear consensus regarding the predominant pathogenic pathways underlying AMD, our work and that of others in neurodegeneration have highlighted that there are redundant complementary cellular death pathways (e.g., apoptosis and receptor interacting protein kinase–regulated necrosis) that may be involved in photoreceptor degeneration. Mechanistic studies have revealed that blocking an individual cellular death pathway may activate another and vice versa.^27^^,^^28^^,^^80^, ^81^, ^82^, ^83^ Furthermore, we have seen that different cellular death pathways may predominate in different cell types.^28^, ^29^, ^30^^,^^81^^,^^82^^,^^84^ Likewise, different upstream stimuli may lead to activation of different cellular death pathways.^31^^,^^32^^,^^85^, ^86^, ^87^ For example, when ROS are the predominant cell death stimulus, ferroptosis (iron-dependent death) is the predominant pathway,^87^, ^88^, ^89^, ^90^, ^91^, ^92^ whereas when tumor necrosis factor alpha and FAS/FAS ligand are the predominant stimuli, apoptosis and receptor interacting protein kinase–dependent necrosis are the predominant pathways.^24^^,^^25^^,^^31^^,^^32^^,^^86^^,^^93^, ^94^, ^95^, ^96^ To make things more complex, infiltrating immune cells also contribute to neurodegeneration via the inflammasome and cross-talk with several other pathways.^80^^,^^85^^,^^97^, ^98^, ^99^, ^100^, ^101^, ^102^, ^103^, ^104^, ^105^, ^106^ On the basis of these findings, we speculate that successful future approaches must target multiple pathways; optimal neuroprotection may require inhibition of upstream and downstream targets simultaneously because cellular death activation is stimulus dependent. This multifactorial approach is not new in medicine. In many other diseases, such as cancer, human immunodeficiency virus, and tuberculosis, combinatorial therapies are the rule rather than the exception.^107^, ^108^, ^109^ The key challenges surrounding neuroprotection as a strategy for treating AMD are summarized in Table 3.

**Table 3:** Key Challenges Surrounding Neuroprotection for AMD

1. Neuroprotection strategies for AMD will likely have value for slowing photoreceptor degeneration and preventing blindness but must be targeted for the right patients at the optimal disease stage to achieve maximal success.
2. Multiple redundant and complementary cellular death pathways are involved in the pathogenesis of AMD and other retinal degenerative diseases.
3. The dominant cellular death pathways differ by retinal cell type.
4. Differing inciting stimuli trigger different downstream cellular death pathways.
5. Optimal neuroprotection may require inhibition of multiple upstream or downstream targets to achieve a clinically significant and durable clinical effect.

AMD = age-related macular degeneration.

## Conclusions

Age-related macular degeneration is a significant cause of blindness worldwide. Neuroprotection has emerged as a new approach for preventing photoreceptor degeneration and subsequent blindness. Although clinical studies to date have not yielded significant results, neuroprotection holds promise for prevention of vision loss caused by photoreceptor death. More research and different approaches are necessary before neuroprotection can emerge as the new frontier for AMD. We remain hopeful that as we gain a more complete understanding of the complexity of AMD, successful clinical results will emerge in the form of novel neuroprotection strategies to treat patients who have AMD and similar conditions.
